# ﻿*Rhynchosporamesoatlantica* (Cyperaceae), an imperiled new species of beaksedge from eastern U.S.A.

**DOI:** 10.3897/phytokeys.236.111271

**Published:** 2023-12-01

**Authors:** Amanda Treher Eberly, Robert F. C. Naczi

**Affiliations:** 1 NatureServe, 2550 South Clark Street, Suite 930, Arlington, VA 22202, USA NatureServe Arlington United States of America; 2 New York Botanical Garden, 2900 Southern Blvd., Bronx, NY 10458-5126, USA New York Botanical Garden New York United States of America; 3 Department of Botany, MRC-166, National Museum of Natural History, Smithsonian Institution, P.O. Box 37012, Washington, D.C. 20013-7012, USA National Museum of Natural History, Smithsonian Institution Washington, D.C. United States of America

**Keywords:** Mid-Atlantic, morphometric analysis, *Rhynchosporafilifolia*, *Rhynchosporaharperi*, *Rhynchospora* section *Fuscae*, sedge

## Abstract

*Rhynchosporamesoatlantica***sp. nov.** (Cyperaceae) is described, illustrated, and compared with morphologically similar species. *Rhynchosporamesoatlantica* is known only from southern Delaware, southeastern Maryland, and southern New Jersey, all within the Mid-Atlantic region of the U.S.A. It inhabits sunny, wet margins of natural, shallow, nutrient-poor, seasonal ponds of the Coastal Plain. Narrow leaf blades; fruits obpyriform in outline; faces of mature fruits possessing a central, pale, well-demarcated disk; and fruit tubercle margins denticulate for most of their lengths indicate *R.mesoatlantica* is most similar to *R.filifolia* and *R.harperi*. *Rhynchosporamesoatlantica* is unique in its fruit dimensions, scales intermediate in length between *R.filifolia* and *R.harperi*, and relatively long fruit stipe. The NatureServe rank of Critically Imperiled and the IUCN rank of Endangered appear warranted for *R.mesoatlantica* because only six populations are known to be extant, most quite small and isolated; all populations occur within a small geographic area; populations have declined; and serious threats confront the survival of the species.

## ﻿Introduction

*Rhynchospora* Vahl section Fuscae (C.B.Clarke ex Gale) Kük., as circumscribed by Gale (1944) and Kral (1996), is a group of sedges characterized by perianth bristles antrorsely barbed, fruit bodies widest in distal half and with smooth faces, and tubercle margins denticulate. Six species belong to *Rhynchospora* section *Fuscae: R.crinipes* Gale, *R.curtissii* Britton, *R.filifolia* A.Gray, *R.fusca* (L.) W.T.Aiton, *R.harperi* Small, and *R.pleiantha* (Kük.) Gale. These species occur from eastern North America south to northeastern South America, with *R.fusca* also occurring in Europe. The center of diversity is the southeastern U.S.A.

The most recent phylogenetic analysis that included members of RhynchosporasectionFuscae indicated the section was polyphyletic (Buddenhagen et al. 2017). This analysis indicated the four included members of Rhynchosporasect.Fuscae (*R.curtissii*, *R.filifolia*, *R.fusca*, and *R.pleiantha*) belonged to three clades separated from each other by multiple clades composed of species from other sections of *Rhynchospora*, e.g. *R.ciliaris* (Michx.) C. Mohr, *R.fascicularis* (Michx.) Vahl, and *R.lindeniana* Griseb. However, this analysis is based on DNA sequence data from only one marker, *trnL/F*. Future analyses with more ample taxon and molecular sampling will be necessary to settle questions of monophyly of RhynchosporasectionFuscae and relationships of taxa within it.

Several previous authors have included Delaware and Maryland within the range of *R.harperi* (Kral 1996, 2002; LeBlond 1997; McMillan 2007; McAvoy 2013; Maryland Natural Heritage Program 2016, 2021; Knapp and Naczi 2021). In addition, McMillan (2007) included New Jersey within the range of this species. Both Kral (1996: 396) and LeBlond (1997: 278) reported northern plants (from Delaware and Maryland) resembled *R.filifolia* in aspect, but had fruits resembling *R.harperi* plants from the southeastern U.S.A. These authors contended that the northern plants fit within the concept of *R.harperi* and included them within this species.

Among plants previously identified as *Rhynchosporaharperi*, we observed substantial differences between plants of the Mid-Atlantic (Delaware, Maryland, and New Jersey) and plants from farther south, including characters not noted by previous authors. Our observations led us to hypothesize that the Mid-Atlantic plants were a species distinct from *R.harperi*. We tested this hypothesis with field work, herbarium work, and morphometric analyses. The purpose of this paper is to report our results, which supported our hypothesis. Accordingly, we also describe the new species *Rhynchosporamesoatlantica*.

## ﻿Materials and methods

We studied the morphology, geography, and ecology of *Rhynchospora* through herbarium work and field work. For herbarium work, we borrowed specimens from, or studied specimens during, visits to DOV, GA, GH, MO, NCU, NY, PH, US (abbreviations as in Thiers 2023). We directly examined all specimens cited in this paper.

The measurements we report in this paper are ones we made directly from specimens. For morphometric analyses, we selected a representative set of 68 specimens of *Rhynchosporafilifolia* and *R.harperi* to measure. We chose mature, ample specimens from throughout the geographic ranges of these species that exhibited their full range of morphologic variation. Among these specimens, we measured type specimens. All measured specimens are from different populations. We considered populations to be different if their localities are at least 1 km apart and separated by unsuitable habitat. For each of these measured specimens (Appendix 1), we measured eight continuous characters and calculated one ratio of measured characters (Table 1). The characters we studied morphometrically are those suggested to be diagnostic for species and infraspecific taxa by previous authors (Gale 1944; Kükenthal 1950; Kral 2002; McMillan 2007; Naczi and Moyer 2016; Ciafré and Naczi 2022), as well as additional ones we suspected to be diagnostic based on our observations from field and herbarium work.

**Table 1.** Morphologic characters, with their abbreviations, measured on herbarium specimens of *Rhynchospora*.

1. SPKLTL	spikelet length, measured from base of lowest scale or its scar to apex of distalmost scale
2. SCLL	scale length, measured for scale from middle of spikelet, from its base to its apex, including awn when present
3. FRL	fruit length, measured from base of fruit to apex of tubercle
4. FRW	fruit width, measured at widest point
5. STPL	stipe length, measured from base of fruit to point at which it widens
6. LBRL	longest perianth bristle length, measured from base of fruit to apex of bristle
7. FRBDYL	fruit body length, measured from base of fruit to summit of fruit body
8. TL	tubercle length, measured from summit of fruit body to apex of tubercle
9. RTLFRL	tubercle length/fruit length

We plotted measurements of characters that were not highly correlated with each other (r < 0.7, thus probably not genetically redundant) in order to detect groups within the morphometric dataset. We then used ANOVA to test for differences among the groups. We performed all statistical analyses with SYSTAT version 11 (SYSTAT Software 2004).

Field work furnished geographic and ecologic data. To determine the geographic range of *Rhynchosporamesoatlantica*, we used specimen collection data to map all known occurrences. For each population of *Rhynchospora* that we studied in the field, we noted vascular plant taxa growing in close association with the target species. We considered closely associated plant taxa to be those growing within 10 meters of *R.mesoatlantica*.

## ﻿Results

Within RhynchosporasectionFuscae, *R.crinipes*, *R.curtissii*, *R.filifolia*, and *R.harperi* comprise a group characterized by four features: cespitose habit, fruit body compressed, mature fruit body with well-demarcated pale disk on center of each face, and fruit with tubercle margins denticulate for most of their lengths (both proximally and distally). The other members of Rhynchosporasect.Fuscae, *R.fusca* and *R.pleiantha*, have a long-creeping rhizomatous habit, fruit bodies biconvex, mature fruit body uniformly brown, and tubercles denticulate only in the proximal half.

*Rhynchosporacurtissii* is quite distinctive in having the perianth bristles long [longest perianth bristle (3.0–)3.5–4.2 mm long in *R.curtissii* vs. 1.5–2.7(–3.1) mm long in other members of Rhynchosporasect.Fuscae]. In addition, in *R.curtissii*, the fruit body is narrowly oblong-obovate in outline (vs. obovate or obpyriform in other members of Rhynchosporasect.Fuscae).

*Rhynchosporacrinipes* is distinctive in having relatively wide leaves (widest leaf blade per plant 2.2–3.8 mm wide in *R.crinipes* vs. 0.6–1.9 mm wide in other members of RhynchosporasectionFuscae). Also, *R.crinipes* has relatively long fruits [fruits, including tubercles, 2.6–2.9 mm long in *R.crinipes* vs. 1.5–2.6(–2.8) mm long in other members of Rhynchosporasect.Fuscae] with a long stipe (stipe 0.45–0.83 mm long in *R.crinipes* vs. 0.11–0.38 mm long in other members of Rhynchosporasect.Fuscae).

**Figure 1.**
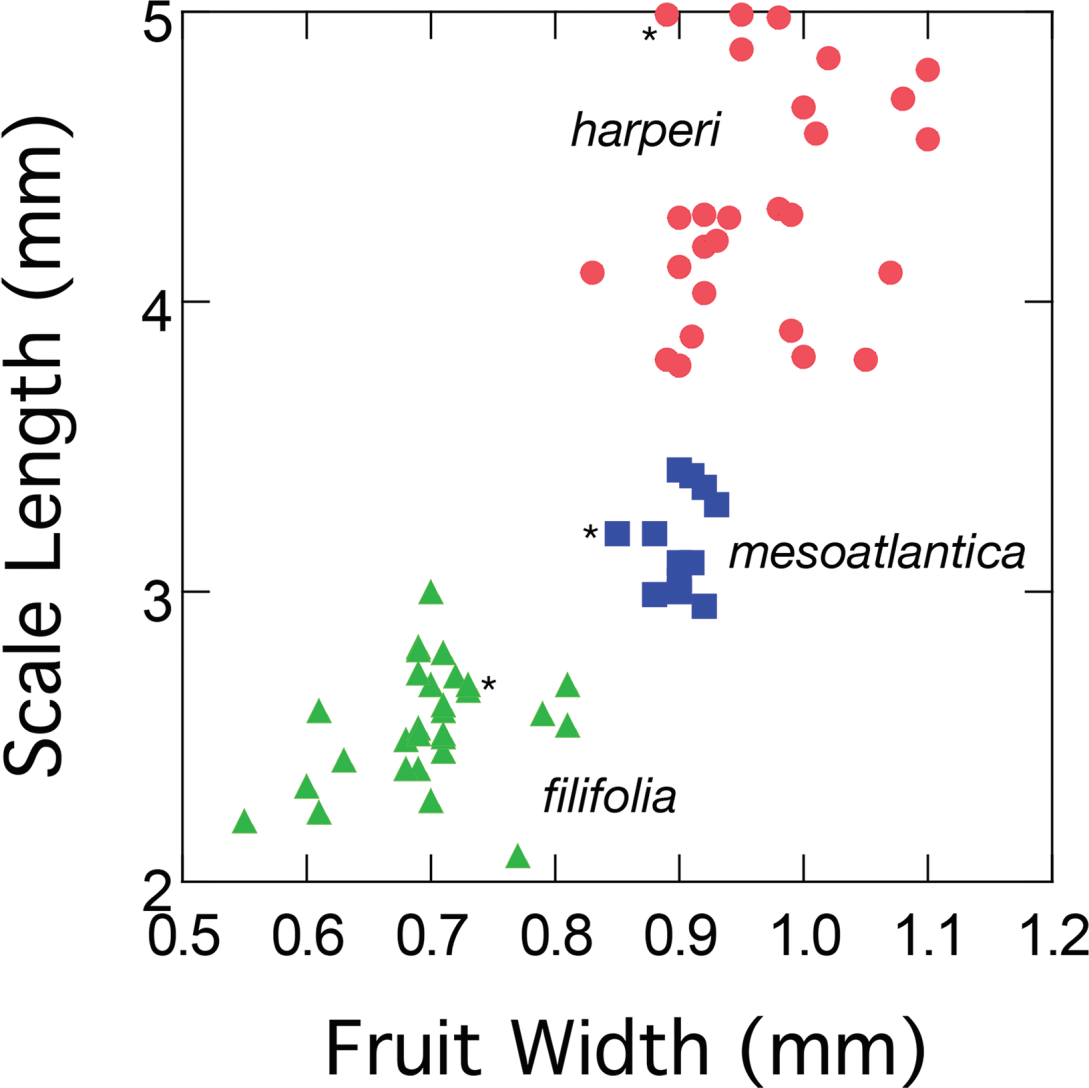
Scale length vs. fruit width for *Rhynchosporafilifolia*, *R.harperi*, and *R.mesoatlantica*. Asterisked symbols indicate lectotype of *R.filifolia*, holotype of *R.harperi*, and holotype of *R.mesoatlantica*.

The remaining species of Rhynchosporasect.Fuscae, *R.filifolia* and *R.harperi*, are more similar to each other than they are to other species of the section. A plot of scale length (SCLL) vs. fruit width (FRW) for these two species reveals three clusters of points (Fig. 1). Inclusion of measurements from type specimens enables identification of these groups. These clusters correspond to *R.filifolia*, another to *R.harperi*, and a third corresponds to plants from Delaware, Maryland, and New Jersey that we propose as a new species and describe below as *R.mesoatlantica*. For this plot, all of the clusters of points are separate and non-overlapping.

**Figure 2.**
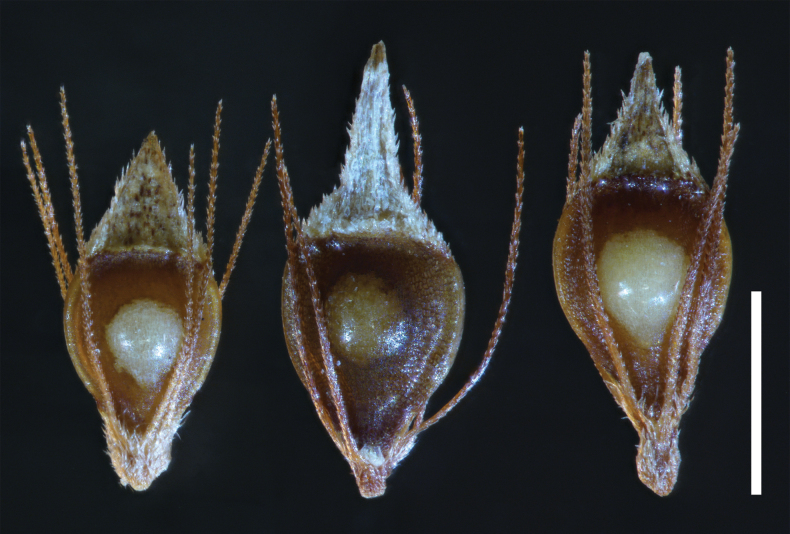
Representative mature fruits of *Rhynchospora* species. Left to right: *Rhynchosporafilifolia* [*Naczi 12060A & Treher* (NY)], *R.harperi* [*Naczi 16347* (NY)], and *R.mesoatlantica* [*Naczi 12060 & Treher* (NY)]. Scale bar: 1.0 mm.

Most characters measured are statistically significantly different among *Rhynchosporafilifolia*, *R.harperi*, and *R.mesoatlantica* (Table 2). The characters that best differentiate the species, judging by ANOVA *F* scores, are scale length (SCLL), spikelet length (SPKLTL), tubercle length (TL), fruit width (FRW), and fruit length (FRL), in descending order of discriminatory power. For every one of these five characters, *R.filifolia* has the lowest values, *R.harperi* has the highest values, and *R.mesoatlantica* has intermediate values. For measurements of one character, stipe length (STPL), *R.mesoatlantica* has greater values than both *R.filifolia* and *R.harperi* (Table 2, Fig. 2). For fruit body length (FRBDYL) and longest perianth bristle length (LBRL), *R.mesoatlantica* and *R.harperi* have very similar measurements, and both have larger values than in *R.filifolia*.

**Table 2.** Means ± 1 SD and ranges for morphologic characters measured for *Rhynchospora*. Character abbreviations correspond to those in Table 1. All measurements are in millimeters. *N* = sample size. Within a row, means with different superscripts differ significantly (ANOVA, *P* < 0.01).

Character	*R.filifolia* (*N* = 29)	*R.harperi* (*N* = 27)	*R.mesoatlantica* (*N* = 12)	ANOVA *F*
1. SPKLTL	3.3^a^ ± 0.44 (2.5–4.3)	5.9^b^ ± 0.58 (5.0–7.2)	4.2^c^ ± 0.31 (3.6–4.7)	220
2. SCLL	2.5^a^ ± 0.20 (2.1–3.0)	4.3^b^ ± 0.40 (3.8–5.0)	3.2^c^ ± 0.17 (3.0–3.4)	260
3. FRL	1.7^a^ ± 0.11 (1.5–1.9)	2.4 ^b^± 0.19 (2.1–2.8)	2.2^c^ ± 0.055 (2.1–2.3)	140
4. FRW	0.70^a^ ± 0.058 (0.55–0.81)	0.97^b^ ± 0.071 (0.83–1.1)	0.90^c^ ± 0.022 (0.85–0.93)	150
5. STPL	0.24^a^ ± 0.046 (0.16–0.34)	0.26^a^ ± 0.044 (0.20–0.35)	0.33^b^ ± 0.030 (0.29–0.38)	21
6. LBRL	1.8^a^ ± 0.20 (1.5–2.3)	2.3^b^ ± 0.29 (1.5–2.9)	2.2^b^ ± 0.10 (2.0–2.4)	31
7. FRBDYL	1.2^a^ ± 0.098 (1.1–1.4)	1.5^b^ ± 0.15 (1.2–1.8)	1.6^b^ ± 0.067 (1.5–1.7)	56
8. TL	0.51^a^ ± 0.052 (0.39–0.62)	0.85^b^ ± 0.087 (0.70–0.99)	0.63^c^ ± 0.050 (0.56–0.70)	180
9. RTLFRL	0.29^a^ ± 0.028 (0.24–0.34)	0.36^b^ ± 0.028 (0.30–0.45)	0.29^a^ ± 0.022 (0.26–0.32)	48

We observed syntopy of *Rhynchosporafilifolia* and *R.mesoatlantica* at one site in Sussex County, Delaware. At this site, we discovered the two species growing within 5 meters of each other (*R.filifolia: Treher 84a*, *Naczi 12060A*; *R.mesoatlantica: Treher 84*, *Naczi 12060*).

## ﻿Discussion

Comparative morphology and morphometric analyses support the hypothesis that *R.mesoatlantica* is distinct from all other *Rhynchospora* species. Multiple morphologic features clearly place *Rhynchosporamesoatlantica* in Rhynchosporasect.Fuscae: perianth bristles antrorsely barbed, fruit bodies widest in distal half and with smooth faces, and tubercle margins denticulate. Additional features place *R.mesoatlantica* as most similar to *R.filifolia* and *R.harperi*: habit cespitose, widest leaf blade < 2.0 mm wide, longest perianth bristle < 3.0 mm long, fruit body compressed and with a well-demarcated pale disk on the center of each face, and tubercle margins denticulate for most of their lengths.

Several morphologic characters distinguish *Rhynchosporamesoatlantica* from *R.filifolia* and *R.harperi*. A plot of SCLL vs. FRW provides complete separation of *R.mesoatlantica* from both *R.filifolia* and *R.harperi* (Fig. 1). In addition, ANOVA reveals *R.mesoatlantica* is statistically significantly different from both *R.filifolia* and *R.harperi* in six of the nine characters studied in the morphometric analysis: SPKLTL, SCLL, FRL, FRW, STPL, and TL. Two additional characters distinguish *R.mesoatlantica* from *R.filifolia* (FRBDYL, LBRL), and one other character distinguishes *R.mesoatlantica* from *R.harperi* (RTLFRL). All these diagnostic characters are from fruits, scales, and spikelets.

Syntopy of *Rhynchosporafilifolia* and *R.mesoatlantica* is another line of evidence supporting species status for *Rhynchosporamesoatlantica*. Despite *R.mesoatlantica* growing in close proximity to *R.filifolia*, the two species maintain their morphologic distinctions at the syntopic site, as well as in all known populations. This naturally occurring syntopy provides a strong test of species distinctions for *R.filifolia* and *R.mesoatlantica*. However, the geographic ranges of *Rhynchosporamesoatlantica* and *R.harperi* do not overlap, making syntopy of these two species impossible.

Specimens of *Rhynchosporamesoatlantica* collected prior to our work had been determined as *R.filifolia* or *R.harperi*. Now that we have presented support for species status for *R.mesoatlantica*, we name and describe this species in order to clarify its status and bring attention to it as a species of conservation concern.

### ﻿Taxonomic treatment

#### ﻿ Rhynchospora mesoatlantica

Taxon classificationPlantaePoalesCyperaceae

A.Eberly & Naczi sp. nov.

FDD096FC-5C38-5529-8AA1-02AABA6B03E3

urn:lsid:ipni.org:names:77332119-1

Figs 2 , 3 , 4

##### Type.

U.S.A., Delaware: Sussex County, 2 mi E of Bayard, Assawoman Wildlife Area, 29 Sep 2007, *A. Treher 84 & R. Naczi* (holotype: NY [measured for morphometric analyses]; isotypes: DOV, PH, US).

##### Diagnosis.

*Rhynchosporamesoatlantica* is similar to *R.filifolia* and *R.harperi*, but *R.mesoatlantica* differs by its fruit dimensions, scales intermediate in length between *R.filifolia* and *R.harperi*, and relatively long fruit stipe. In *R.mesoatlantica*, scales are 3.0–3.4 mm long, and tubercles are 0.6–0.7 mm long and 26–32% of fruit length, in contrast to *R.harperi*, which has scales 3.8–5.0 mm long, and tubercles 0.7–1.0 mm long and (30–)33–39(–45)% of fruit length. In *R.mesoatlantica*, scales are 3.0–3.4 mm long, and fruits are 2.1–2.3 mm long and 0.9 mm wide, in contrast to *R.filifolia*, which has scales 2.1–3.0 mm long, and fruits 1.5–1.9 mm long and 0.6–0.8 mm wide. *Rhynchosporamesoatlantica* has fruit stipes 0.29–0.38 mm long, in contrast to *R.filifolia* (0.16–0.34 mm long) and *R.harperi* (0.20–0.35 mm long).

##### Description.

***Culm*** (2–)3–9 dm tall, 0.4–1.2 mm wide at midpoint, erect. ***Leaves*** filiform, flexuous; proximal leaf blades 7–25 cm long, 0.2–0.6 times the culm height, 0.5–0.8 mm wide, margins involute; cauline leaf blades 9–21 cm long, 0.5–1.5 mm wide, margins involute. ***Infructescence*** composed of 1–3 (–4) compound fascicles per culm. ***Fascicles*** hemispheric to occasionally turbinate, 1.0–2.0 cm wide, composed of 5–75 spikelets, branches of subfascicle 0.3–0.9 cm long, 0.2–0.3 mm wide; distalmost fascicle bracts 1–3, 2–13 cm long, 1–1.5 mm wide. ***Spikelets*** 3.6–4.7 mm long, proximal scales 1.5–2 mm long, scales from middle of spike 3.0–3.4 mm long, cinnamon brown with darker brown central nerve. ***Perianth bristles*** 6, the longest per fruit 2.0–2.4 mm long, 0.8–1.1 times as long as fruit (including tubercle), antrorsely barbellate. ***Fruit*** (including the tubercle) 2.1–2.3 mm long, 0.85–0.93 mm wide, bearing persistent perianth bristles; body 1.5–1.7 mm long, obpyriform in outline, surface shiny, smooth, brown or reddish-brown except for whitish and well-demarcated central disk on each face, central disk occupying 0.4–0.8 of fruit width; tubercle with straight or slightly concave margins, 0.56–0.70 mm long, 0.26–0.32 of fruit length, 0.6–0.7 mm wide at base; stipe 0.29–0.38 mm long.

**Figure 3.**
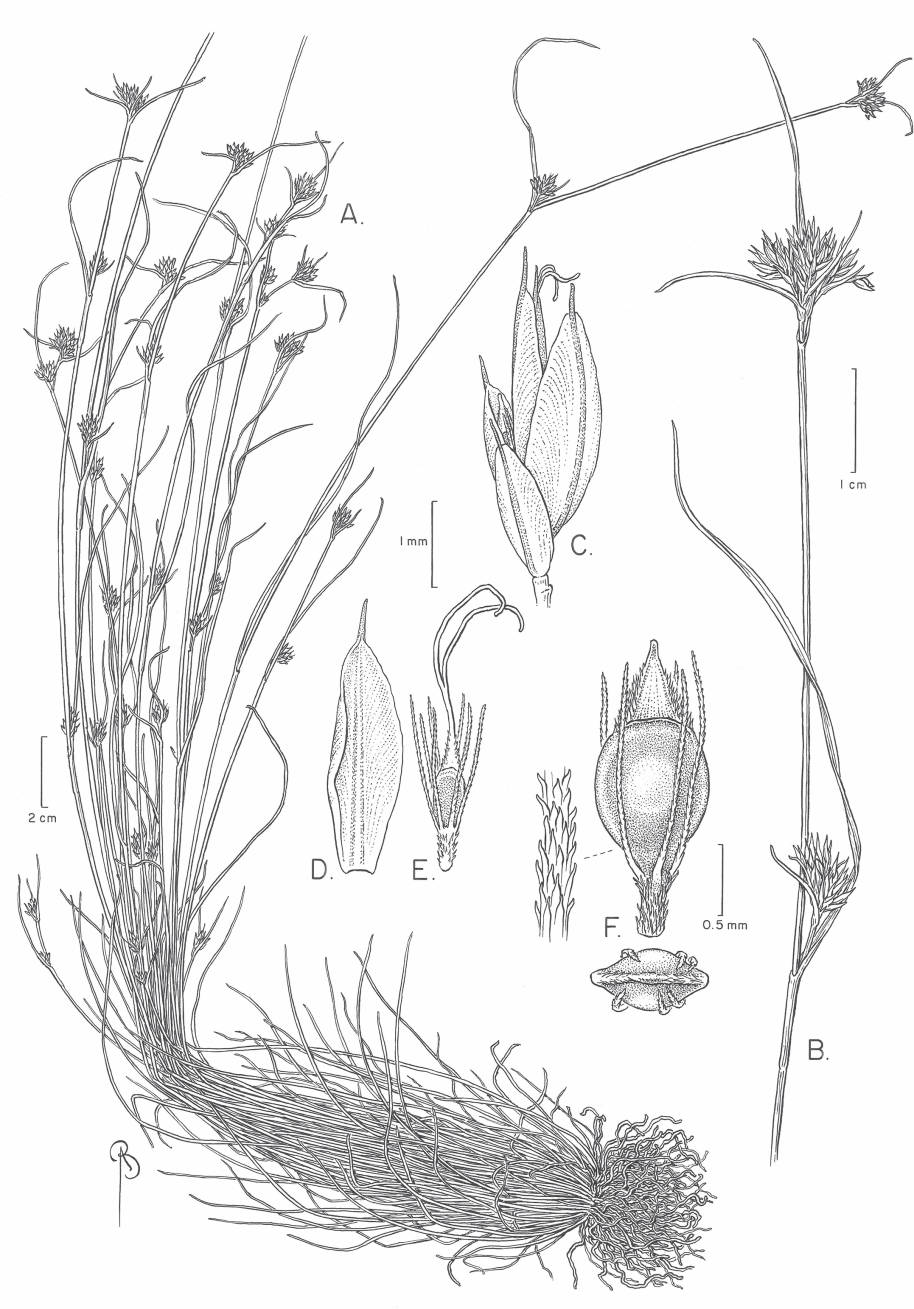
*Rhynchosporamesoatlantica***A** habit **B** distal portion of infructescence **C** spikelet **D** distal scale **E** immature fruit **F** mature fruit, lateral view, with detail of perianth bristle (left) and top view (below). From *Treher 84 & Naczi* (Holotype, NY). Scale bars: 2 cm (**A**); 1 cm (**B**); 1 mm (**C, D, E**); 0.5 mm (**F**).

**Figure 4.**
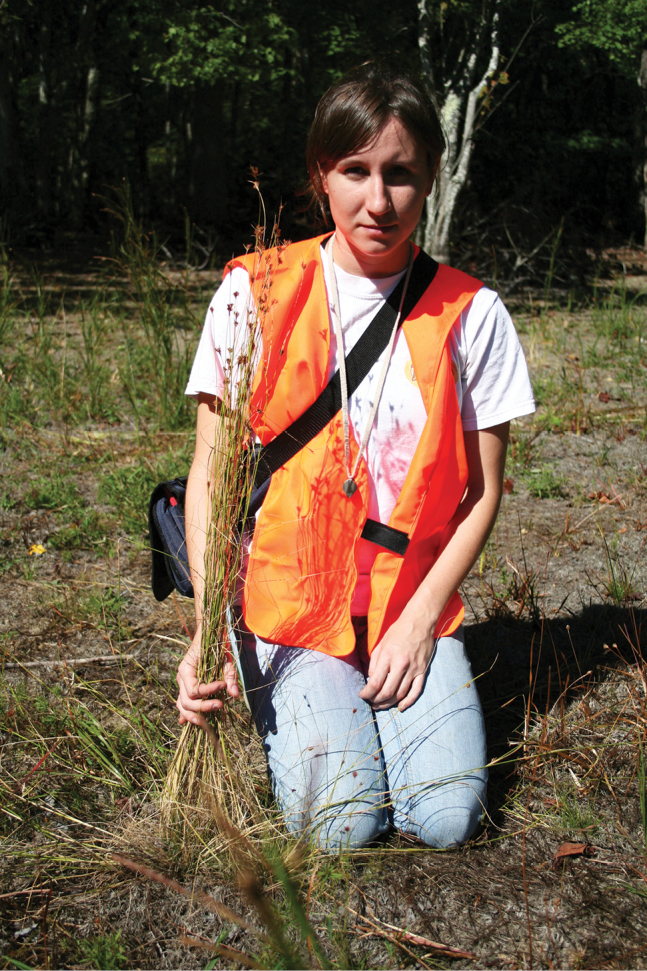
Habit of *Rhynchosporamesoatlantica*. Amanda T. Eberly with *R.mesoatlantica* rooted in habitat at type locality (*Treher 84 & Naczi*).

##### Etymology.

We name *Rhynchosporamesoatlantica* for the Mid-Atlantic region of the U.S.A., the region in which all known populations occur.

##### Geographic distribution.

**Figure 5.**
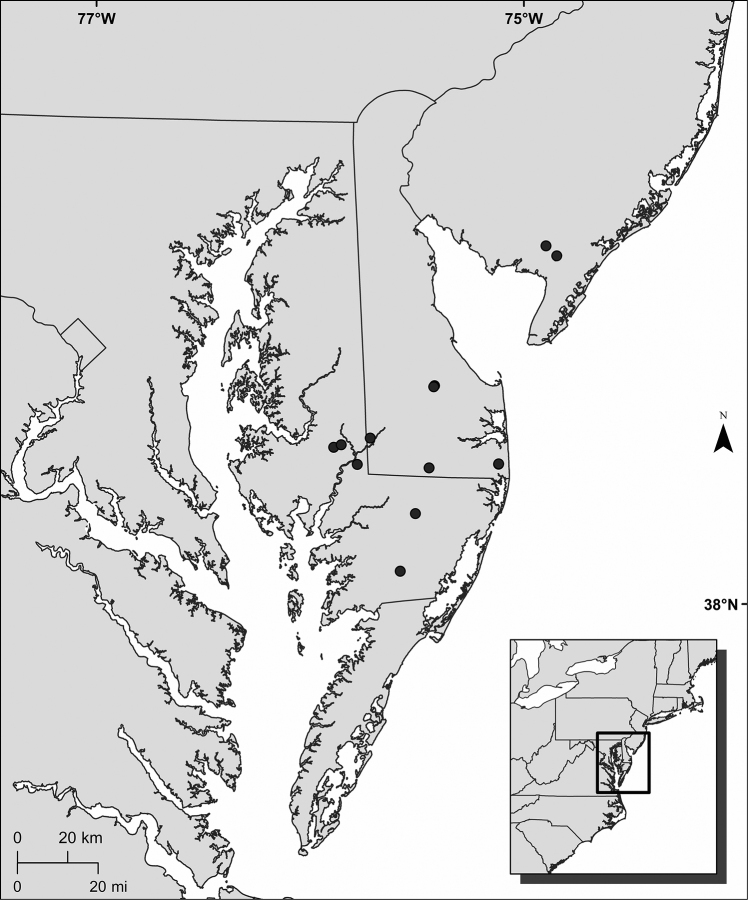
Known geographic distribution of *Rhynchosporamesoatlantica*. Based on all known collections.

*Rhynchosporamesoatlantica* is a narrow endemic of a portion of the Mid-Atlantic U.S.A. (Fig. 5). It is known only from southern New Jersey, southern Delaware, and southeastern Maryland, where it occurs on the Coastal Plain physiographic province. Specimens document its occurrence from a total of 12 populations, each separated by at least 1 km from other populations. Two of the populations in the vicinity of Ellendale, Delaware [E of Ellendale, *Commons s.n.* (PH); S of Ellendale, *McAvoy 6333* (DOV) and later collections] are sufficiently close (3 km apart) that they map as one population (Fig. 5). Other populations are separated by greater distances. The greatest distance separating nearest neighbors among populations (*Moyer G0272* in Cape May County, New Jersey, and *Commons s.n.* in Sussex County, Delaware) is 70 km.

##### Habitat.

**Figure 6.**
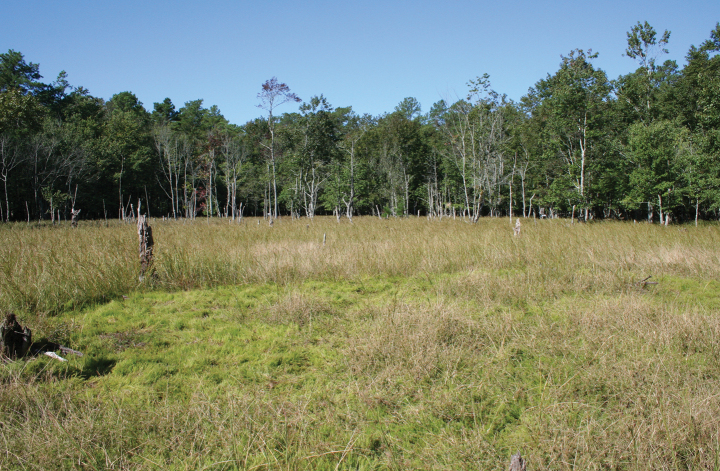
Representative habitat of *Rhynchosporamesoatlantica*. At type locality (*Treher 84 & Naczi*).

*Rhynchosporamesoatlantica* grows on the sunny, moist upper portions of natural, shallow, nutrient-poor, seasonal ponds and depressions with gently sloping shorelines and sandy-peaty soils (Fig. 6). Surrounding these wetlands are dry-mesic forests or pine plantations. Water levels are typically highest in winter and spring, which is characteristic of Coastal Plain ponds (Phillips and Shedlock 1993). By the time of fruiting, the ponds are usually devoid of standing water, and the plants grow in soils that are merely moist. At most sites we visited, natural seasonal fluctuations in water levels were disrupted by extensive ditching and draining that apparently lowered the water table. Drier soils throughout the year have provided favorable growing conditions for woody vegetation, which is slowly overgrowing and shading some of the sites. The least disturbed site had few trees and shrubs (Fig. 6). In the absence of the natural disturbance of fluctuating water levels, management appears necessary to maintain a sunny environment. *Rhynchosporamesoatlantica* may persist vegetatively or in the seed bank during periods of unfavorable conditions, but research is needed to understand its persistence and dormancy.

Close plant associates (those growing within 10 m) of *Rhynchosporamesoatlantica* are *Acerrubrum* L., *Boltoniaasteroides* (L.) L’Hér. (*Treher 75 & Naczi*, DOV), *Cladiummariscoides* (Muhl.) Torr. (*Treher 74 & Naczi*, DOV), *Coelorachisrugosa* (Nutt.) Nash (*Naczi 12056 & Treher*, DOV, PH; *Treher 72 & Naczi*, DOV), Coleataenialongifolia(Torrey)Sorengssp.longifolia, *Dichantheliumspretum* (Schult.) Freckmann (*Naczi 12057 & Treher*, NY, PH), *Eleocharistenuis* Schult., *Hypericumdenticulatum* Walter (*Naczi 12058 & Treher*, DOV), *Juncuscanadensis* J.Gay in Laharpe (*Naczi 12064 & Treher*, NY; *Treher 82 & Naczi*, DOV), *Juncusrepens* Michx. (*Naczi 12062 & Treher*, NY; *Treher 78 & Naczi*, DOV), *Kellochloaverrucosa* (Muhl.) Lizarazu, Nicola, & Scataglini (*Treher 116 & Naczi*, DOV), *Proserpinacapectinata* Lam. (*Treher 79 & Naczi*, DOV), *Rhexiaaristosa* Britton (*Naczi 12065 & Treher*, DOV), *Rhexiavirginica* L. (*Treher 118 & Naczi*, DOV), *Rhynchosporachalarocephala* Fernald & Gale (*Naczi 12086 & Treher*, NY; *Treher 112 & Naczi*, DOV), *Rhynchosporafilifolia* (*Naczi 12060A & Treher*, NY; *Treher 84a & Naczi*, DOV), *Rhynchosporagracilenta* A.Gray (*Treher 113 & Naczi*, DOV), *Rhynchosporainundata* (Oakes) Fernald (*Naczi 12061 & Treher*, DOV), *Saccharumgiganteum* (Walter), *Scleriareticularis* Michx. (*Naczi 12063 & Treher*, NY; *Treher 77 & Naczi*, DOV), *Sclerolepisuniflora* (Walter) Britton, Sterns, & Poggenb. (*Naczi 12059 & Treher*, DOV; *Treher 73 & Naczi*, DOV), and *Sphagnummacrophyllum* Bernh. ex Brid. Pers.

##### Preliminary conservation assessment.

*Rhynchosporamesoatlantica* is at a high risk of extinction due to a restricted geographic range, small number of occurrences, small population sizes, and historic and ongoing declines due to numerous threats. All historic and current populations total 12. Six of the populations have not been seen for over 20 years, despite repeated, more recent surveys at most of the sites. Three of these populations had been documented in the 1990s, yet we could not relocate them. Thus, declines are apparent in number of populations and number of plants. We are sufficiently familiar with some of these sites to identify likely causes for extirpations: habitat destruction for some and, for others, habitat degradation, including changes to hydrology.

Only six populations are known to be extant. Populations are typically small, ranging from 25 to a maximum of 200–300 plants at the population northwest of Belleplain (R. Moyer, pers. comm.). Only three populations contain more than 100 plants. Our estimate of the total number of mature plants present in extant populations is 700.

Five of the six populations known to be extant are in protected areas. Most of these protected areas are state forests that allow resource extraction and consequent habitat alteration.

Most extant and historic occurrences are/were in Coastal Plain ponds in Delaware and Maryland, one of the most threatened habitats on the Delmarva Peninsula and host to many rare species (McAvoy and Bowman 2002). Most of these ponds and surrounding forests are highly degraded due to direct and indirect anthropogenic impacts. Land-use changes resulting in habitat fragmentation, conversion of forest to pine plantations, destructive forestry practices like clear-cutting, and hydrologic alterations due to extensive ditching and draining are among the threats contributing to past and ongoing declines (McAvoy and Bowman 2002). Quantifying declines in *Rhynchosporamesoatlantica* is challenging; the historic record is sparse, with only four populations documented prior to 1990. Landscape changes are evident throughout the Delmarva Peninsula, including the extent of ditching and draining. In Delaware alone, there are over 2,000 miles of ditches intended to redirect normal water flows across the land and sustain productive agricultural lands (DE DNREC 2023). Unfortunately, these ditches negatively impact natural plant communities hosting *R.mesoatlantica* by interrupting seasonal water-level fluctuations that suppress woody vegetation. Habitat restoration with ongoing maintenance, especially for natural hydrologic cycles, appears to be warranted at most sites, including those on public lands.

Also noteworthy is the fact that *R.mesoatlantica* plants usually occupy only a portion, and often a small portion, of the Coastal Plain ponds that host this species. For example, the area of one pond is 0.008 km^2^ (8,000 m^2^), yet plants of *R.mesoatlantica* occupy only 0.004 km^2^ (4,000 m^2^) of the pond. Our estimate of the area occupied by all known *R.mesoatlantica* populations, historic and extant, is 0.031 km^2^ (31,000 m^2^). For *R.mesoatlantica* populations known to be extant, our estimate of area occupied is 0.017 km^2^ (17,000 m^2^).

Due to decades-long recognition of Coastal Plain ponds as centers of rare plant diversity (e.g., Hirst 1983; Boone et al. 1984; McAvoy and Bowman 2002) and our own extensive field efforts to rediscover formerly documented populations of *Rhynchosporamesoatlantica* and discover new ones, we regard the likelihood of discovery of new populations as low. Simply, most Coastal Plain ponds within the geographic range of *R.mesoatlantica* have been botanically explored, many very extensively during multiple years and multiple seasons.

We recommend a NatureServe Global Rank of Critically Imperiled (G1, Faber-Langendoen et al. 2012) for *Rhynchosporamesoatlantica*, based on considerations of rarity, threats, and trends (Master et al. 2012). There are 12 known occurrences (6 historic and 6 extant), a Range Extent (Extent of Occurrence, EOO) of 4,495 km^2^, and an Area of Occupancy (AOO) of 44 km^2^. Threat impact is estimated at very high, and short-term trends and long-term trends are estimated to be at least 10% and 40%, respectively, based on declines in AOO, population size, and number of occurrences.

As a preliminary assessment, we consider the IUCN category Endangered (IUCN Standards and Petitions Committee 2022) to apply to *Rhynchosporamesoatlantica* for the following reasons: EOO of 4,495 km^2^ is < the 5,000 km^2^ threshold (B1); AOO of 44 km^2^ is < the 500 km^2^ threshold (B2); and we have observed continuing decline in AOO, habitat quality, and number of populations (Bb). Tentatively, we assess the metapopulation as severely fragmented since at least 50% of the populations are isolated and small (< 50 plants) and occurring in a very rare and localized habitat surrounded by unsuitable habitats and with limited capacity for dispersal between distant extant populations 11–70 km apart (Ba).

Due to the severity of conservation threats, few known extant populations, small population sizes, and apparent necessity of human-mediated intervention to maintain habitats, we recommend *Rhynchosporamesoatlantica* for protection under the U.S.A. Endangered Species Act.

##### Additional specimens examined.

(* = specimen measured for morphometric analyses)—**U.S.A. Delaware**: Sussex Co., **Population 1**: E of Bayard, 26 Sep 1986, *Hirst 459* (DOV); Assawoman Wildlife Area, 8 Sep 1991, *McAvoy s.n.* (US); E of Bayard, Assawoman Wildlife Area, 31 Nov 1991, *Hirst 449* (DOV); Assawoman Wildlife Area, 22 Nov 1992, *McAvoy 243* (DOV); Assawoman Wildlife Area, 1.7 mi E of Bayard, 11 Nov 1993, *Hirst 309* (DOV); Assawoman Wildlife Area, 16 Aug 1995, *McAvoy 1234* (DOV); 2 mi E of Bayard, Assawoman Wildlife Area, 29 Sep 2007, *Naczi 12060 & Treher* (MO, NY, PH). **Population 2**: E of Ellendale, 17 Aug 1899, *Commons s.n.* (PH*). **Population 3**: S of Ellendale, Redden State Forest tract, N side of Saw Mill Road, E of Spicer Road, 29 Oct 2007, *6333 McAvoy* (DOV); Redden State Forest, N side of Saw Mill Road, E of Spicer Road, SE of Ellendale, 5 Aug 2008, *McAvoy 6417* (DOV); 4.5 mi W of Milton, 25 Sep 2008, *Treher 373* & *McAvoy* (DOV*); S of Ellendale, N side of Saw Mill Road, 21 Aug 2013, *McAvoy 7220* (NY). **Population 4**: 1.8 mi NNE of Whitesville, 12 Sep 1992, *Hirst 415 & Wilson* (DOV); 1.8 mi NNE of Whitesville, 12 Sep 1992, *Hirst 416 & Wilson* (DOV); 1.5 mi N of Whitesville, 27 Jul 1993, *Hirst 409 & Wilson* (DOV*); SE of Pepperbox, 30 Jul 1997, *McAvoy 2765* (DOV). **Population 5**: 1.8 mi SW of Woodland, 28 Aug 1993, *Hirst 410 & Wilson* (DOV*). **Maryland**: Dorchester Co., **Population 6**: 1.7 mi NW of Reids Grove, 21 Aug 1998, *Hirst 1198 & Wilson* (DOV); 0.2 mi SE of junction of Centennial and Kraft Roads, 21 Aug 1998, *Hirst 1200 & Wilson* (DOV); NW of Reids Grove, 28 Aug 1998, *McAvoy 3994* (DOV); 3.4 mi SW of Brookview, 1.8 mi NW of Reids Grove, 29 Aug 1998, *Hirst 1208 & Wilson* (DOV); 1.8 mi NW of Reids Grove, 3.4 mi SW of Brookview, 29 Aug 1998, *Hirst 1209 & Wilson* (DOV); 3.3 mi SW of Brookview, 1 Oct 2008, *Treher 377 & Knapp* (DOV*). **Population 7**: 1.5 mi SW of Brookview, 20 Sep 1997, *Hirst 1189 & Wilson* (DOV); W of Brookview, 4 Oct 1997, *McAvoy 3160* (DOV*); S of Brookview, 28 Aug 1998, *McAvoy 4002* (DOV); 1.4 mi SSW of Brookview, 2.0 mi NNE of Reids Grove, 29 Aug 1998, *Hirst 1207 et al.* (DOV). Wicomico Co., **Population 8**: NE of Mardela Springs, 17 Sep 2000, *Hirst 1234 & Wilson* (DOV*). **Population 9**: 1.5 mi W of Wango, 2 Oct 2007, *Treher 110 & Naczi* (DOV), 1.5 mi W of Wango, SW of junction of Twilleys Bridge Road and Fooks Road, 2 Oct 2007, *Naczi 12087 & Treher* (NY); S of Twilley’s Bridge Road, W of Powellville, 30 Sep 2014, *McAvoy 7465* (DOV*). Worcester Co., **Population 10**: 5 mi N of Pocomoke, Pocomoke State Forest, 6 Oct 1984, *Hirst 418* (DOV*); N of Pocomoke, Pocomoke State Forest, 22 Aug 1986, *Hirst 439* (DOV). **New Jersey**: Cape May Co., **Population 11**: Woodbine, 30 Aug 1900, *S. Brown 4289* (NY, PH*); Between Belleplain and Woodbine, 4 Sep 1960, *B. Hirst s.n.* (PH). **Population 12**: NW Belleplain, 24 August 2015, *R. Moyer G0272* (NY*).

### ﻿Identification key to RhynchosporasectionFuscae

This key is for specimens bearing mature fruits. Measurements of fruit length include the tubercle, but not perianth bristles. Scale length is for scales from middle of spikelets.

**Table:** 

1a	Plants with long-creeping rhizomes; fruit body uniformly brown, biconvex; tubercle margins denticulate only in proximal half	**2a**
2a	Fruit 2.3–3.0 mm long, 0.9–1.3 mm wide; longest perianth bristle 2.7–3.8 mm long	***R.fusca***
2b	Fruit 1.6–2.0 mm long., 0.6–0.8 mm wide; longest perianth bristle 2.1–2.8 mm long	***R.pleiantha***
1b	Plants cespitose; fruit body with pale disk on center of each face, compressed; tubercle margins denticulate for most of their lengths	**3a**
3a	Fruit body narrowly oblong-obovate in outline; longest perianth bristle (3.0–)3.5–4.2 mm long	***R.curtissii***
3b	Fruit body obovate or obpyriform in outline; longest perianth bristle 1.5–2.7(–3.1) mm long	**4a**
4a	Widest leaf blade per plant 2.2–3.8 mm wide; fruit 2.6–2.9 mm long; fruit stipe 0.5–0.8 mm long	***R.crinipes***
4b	Widest leaf blade per plant 0.6–1.9 mm wide; fruit 1.5–2.6(–2.8) mm long; fruit stipe 0.1–0.4 mm long	**5a**
5a	Spikelet 5.0–7.2 mm long; scale 3.8–5.0 mm long; tubercle 0.7–1.0 mm long, (30–)33–39(–45)% of fruit length	***R.harperi***
5b	Spikelet 2.5–4.7 mm long; scale 2.1–3.4 mm long; tubercle 0.4–0.7 mm long, 24–34% of fruit length	**6a**
6a	Scale 2.1–3.0 mm long; fruit 1.5–1.9 mm long, 0.6–0.8 mm wide; fruit stipe 0.2–0.3 mm long	***R.filifolia***
6b	Scale 3.0–3.4 mm long; fruit 2.1–2.3 mm long, 0.9 mm wide; fruit stipe 0.3–0.4 mm long	***R.mesoatlantica***

## Supplementary Material

XML Treatment for Rhynchospora mesoatlantica

## ﻿Additional information

### Author ORCIDs

Amanda Treher Eberly https://orcid.org/0009-0007-3360-7393

Robert F. C. Naczi https://orcid.org/0000-0002-3985-0059

### Data availability

All of the data that support the findings of this study are available in the main text and Appendix 1.

## Appendix

Selected Specimens Examined of *Rhynchosporafilifolia* and *R.harperi*. Asterisked specimens are those measured for morphometric analyses.

***Rhynchosporafilifolia***—**BELIZE. Belize District**: ca. 6 mi SE of La Democracia, along Coastal Highway, ca. 6 mi SE of its junction with Western Highway, 29 Nov 2005, *Naczi 11210 et al.* (BRH, DOV*, NY); 1.6 air mi N of junction of Old Northern Highway and Northern Highway, 0.15 mi E of Old Northern Highway, 15 Mar 2008, *Treher 176 & Gibson* (DOV). **Toledo District**, 6.8 mi NNE of Medina Bank, 2.2 mi S of southern boundary of Belize Foundation for Research and Environmental Education (BFREE), Deep River Forest Reserve, 25 Mar 2006, *Naczi 11315* (BRH, DOV*, NY). **CUBA. [Isla de la Juventud Municipality**]: Vivijagua Savanna, 28–29 Feb 1916, *Britton 15018 et al.* (NY*, US). **Pinar Del Rio Province**: Herradura, 2 & 4 Dec 1904, *Baber & Abarca 4195* (NY*, US). **MEXICO. Tabasco**: km 44.4 rumbo de Huimanguillo a Francisco Rueda, 6 Aug 1979, *Cowan 2237* (NY*). **NICARAGUA. Comarca del Cabo**: Puente Pozo Azul, Kornuk Creek near Bilwaskarma, 14 Mar 1971, *Svenson 4758* (NY*). **U.S.A. Alabama**: Covington Co., Route 7, ca. 9 mi S of Red Level and 3 mi S of Loango, 20 Jun 1967, *Clark 14462* (NCU*). Houston Co., Route 4, ca. 4 mi W Chattahoochee State Park entrance, 5 Jun 1972, *Kral 47253* (NCU*). **Delaware**: Sussex Co., East of Bayard, Assawoman Wildlife Management Area, 14 Sep 1998, *Hirst 1221* (DOV*), 2 mi E of Bayard, Assawoman Wildlife Area, 29 Sep 2007, *Naczi 12060A & Treher* (NY), *Treher 84a & Naczi* (DOV). **Florida**: Bay Co., NW of Panama City, 3.55 mi E of route 79 on route 388, 27 Aug 2000, *Abbott 13938 & Carlsward* (DOV*). Duval Co., 1–2 mi ENE of Bryceville, E of route 301, Cary State Forest, 2 Jun 2000, *Anderson 19290* (NY*). Martin Co., SE of Hobe Sound, Jonathan Dickinson State Park, 7 Jul 2008, *Treher 306 et al.* (DOV). Okeechobee Co., Okeechobee Prairie, North of Lake Okeechobee, 1 May 1919, *Small 9093* (NY*). Palm Beach Co., W of Jupiter, N of Indiantown Road/route 706, Hungryland Environmental and Wildlife Area, 7 Jul 2008, *Treher 273 et al.* (DOV). Sarasota Co., Myakka River State Park, 0.25 mi S of State Rd 72, *Treher 317 et al.* (DOV*). Saint Lucie Co., 18 mi E of Okeechobee City, 8 Dec 1919, *Small 9305* (NY*). Wakulla Co., Along route 372, SE of Sopchoppy, 9 Jun 1960, *Godfrey 59702* (NCU*). **Georgia**: Bacon Co., 4 mi E of Nicholls by route 32, 25 Jun 1993, *Kral 82714 & Carter* (NCU, NY*). Bartow Co., 4.8 mi E, 28 degrees S of Adairsville, 14 July 1951, *Duncan 12730* (NY*). Charlton Co., ca. 2 mi SW of Folkston, along W side of route 121, 28 Aug 2001, *Naczi 8768* (DOV*). **Louisiana**: Allen Parish, end of dirt road running S from Parish Road 2-36, 12 Jun 1996, *Sorrie 8904* (NCU*). **Mississippi**: Harrison Co., 3–4 mi N of Biloxi, 24 Jul 1971, *Rogers 6829* (NCU*). Jackson Co., Ocean Springs, 29 Jul 1952, *Demaree 32463* (PH*). **New Jersey**: Cape May Co., Lower Fishing Creek, Oliver’s Bog, 18 Sep 1914, *Brown s.n.* (PH*). **North Carolina**: [No locality, no date], *Curtis s.n.* (Lectotype, designated by Gale [1944: 175]: NY00277848*). Carteret Co., Croatan National Forest, 1.8 mi NE of Ocean, 20 Aug 2008, *Treher 356* (DOV*). **South Carolina**: Dillon Co., 3.5 mi SW of Latta, 11 Jul 1949, *Godfrey SC49004* (PH, NCU, NY*, US). Georgetown Co., 4 mi NW of North Santee, 13 Jun 1957, *Radford 25128* (NCU*). **Texas**: Burleson Co., 4.6 air mi WSW of Caldwell and 2.4 air mi NW of junction of Routes 21 and 908, 7 Jun 1989, *Orzell 10431 & Bridges* (NCU, NY*). Waller Co., Hempstead, 10 Jun 1872, *Hall 717* (US*, NY). V**irginia**: Sussex Co., Airfield Millpond, SW of Wakefield, 11–12 Sep 1945, *Fernald 14908 & Long* (GH, NY, PH*, US).

***Rhynchosporaharperi***—**BELIZE. Belize District**: 1.6 air mi N of junction of Old Northern Highway and Northern Highway, 0.15 mi E of Old Northern Highway, 15 Mar 2008, *Treher 173 & Gibson* (DOV*); ca. 1 mi W of Hattieville, 0.1 mi S of Western Highway, 15 Apr 2008, *Naczi 12266* (BRH, DOV*, NY, US, W); 4.7 km (2.9 mi) NNW of Sand Hill village, 16 Apr 2016, *Naczi 16347* (BRH, NY). **GUYANA.**5°37'5.6"N, 60°40'58.1"W, 491 m, 19 May 2009, *Wurdack 5101 et al.* (NY, US*). **U.S.A. Alabama**: Baldwin Co., Gulf Shores State Park, NE of Little Lake, 24 Sep 1996, *Sorrie 9050 & LeBlond* (GH, NCU*). **Florida**: Franklin Co., 0.4 mi W of route 65, Apalachicola National Forest, generally S of Sumatra, 14 Jul 1989, *Godfrey 83360 & Gholson* (GA*, GH, NY); by route 65, 2.2 mi N of junction US route 98, 3 Jul 1993, *Kral 82800* (GA, GH, MO*, NY, US); 2 mi drive E of route 65 along S side of Buck Siding Road, 14 Jul 1988, *Anderson 11611* (MO, NY*). Gulf Co., By Florida route 71, 6–7 mi S of Wewahitchka, 20 Jul 1993, *Kral 82854 & Moore* (GH, MO, NY*); 4.3 mi drive S of route 22, 5.5 air mi SW of Wewahitchka, 20 Jul 1989, *Anderson 12*,*170* (MO, NY*). Liberty Co., By route 65, ± 5 mi N of Sumatra, 26 Jul 1993, *Godfrey 84647* (GA*, GH, MO, NY, US); E of route 65 a few mi NE of Wilma, 10 Jul 1992, *Anderson 13*,*706* (NY*). Martin Co., S side Stuart off US route 1, 18 Sep 1973, *Kral 51780* (MO*); *Hypericum*-*Taxodium* pond by Willoughby Ave., 0.25 mi. N jct. county route 722, 8 Jul 1994, *Kral 83706* (MO, NCU*, NY); SE of Hobe Sound, Jonathan Dickinson State Park, 7 Jul 2008, *Treher 307 et al.* (DOV*). Palm Beach Co., W of Jupiter, N of Indiantown Road/route 706, Hungryland Environmental and Wildlife Area, 7 Jul 2008, *Treher 272 et al.* (DOV*). Polk Co., 2.5 air mi SW of Hesperides, 11 May 1991, *Orzell 16650 & Bridges* (GA, NY*, US); 1.7–2.0 mi air mi N of junction FL 630, ca. 1 air mi SW of Lake Weohyakapka, 12 May 1991, *Orzell 16666 & Bridges* (NY*). Sarasota Co., 0.7 mi S of Myakka River State Park and Manatee County line, 0.3 mi S of FL 72 at a point ca. 5 mi E of Myakka River bridge, 9 May 1991, *Orzell 16565 & Bridges* (NY*, US); Myakka River State Park, 0.25 mi S of State Rd 72, *Treher 315 et al.* (DOV). Wakulla Co., just E of Sopchoppy, St. Mark National Wildlife Refuge, *Orzell 13967 & Bridges* (MO*). Walton Co., Florida route 20, 1.1 mi E of Bruce, 18 Jul 1995, *Kral 85337* (GH, MO, NY*, US). Washington Co., 0.5–1 mi W of Bay County line by Florida route 20 just E of Ebro, 19 Jul 1993, *Kral 82820 & Moore* (GH, MO, NY*, US). **Georgia**: Long Co., ca. 6 mi NE of Ludowici on W side of US route 82, 3 Nov 1993, *Sorrie 7777 et al.* (GA*, NCU). Pulaski County, ca. 3 mi E of Hawkinsville, 26 June 1902, *Harper 1377* (Holotype: NY00051395*; Isotype: US00087005). Sumter Co., Wet pine barrens, 23 Aug 1900, *Harper 467* (GH, NY*, US). **Mississippi**: Jackson Co., Between route 613 and railroad, 0.25 mi N of Frank Snell Road, 10 Nov 1997, *Sorrie 9632* (NCU). **North Carolina**: Brunswick Co., Hog Branch Ponds Natural Area, 13 Sep 1993, *LeBlond 3623A* (NCU*). Carteret Co., Croatan National Forest, 1.4 mi NE of Ocean, 20 Aug 2008, *Treher 362 & LeBlond* (DOV*). **South Carolina**: Berkeley Co., S of Route 45, S of Honey Hill, 4 Aug 1997, *McMillan 2632* (NCU*).
